# Nebulized versus intravenous morphine titration for the initial treatment of severe acute pain in the emergency department: study protocol for a multicenter, prospective randomized and controlled trial, CLIN-AEROMORPH

**DOI:** 10.1186/s13063-019-3326-3

**Published:** 2019-04-11

**Authors:** Virginie Eve Lvovschi, Justine Joly, Nicolas Lemaire, Maxime Maignan, Pauline Canavaggio, Anne-Marie Leroi, Marie-Pierre Tavolacci, Luc-Marie Joly

**Affiliations:** 1 Emergency Department, Rouen University Hospital, 76031 Rouen, France; 2 Emergency Department, Grenoble University Hospital, Univ. Grenoble Alpes, Inserm, CHU Grenoble Alpes, HP2, Grenoble Alps University, 38000 Grenoble, France; 3 Emergency Department, Pitié-Salpêtrière University Hospital, Assistance Publique-Hôpitaux de Paris (APHP), Paris, France; 4 Normandie Univ, UNIROUEN, INSERM U1073, Rouen, France

**Keywords:** Morphine, Pain, Analgesia, Nebulized, Randomised controlled trial, Single blind

## Abstract

**Background:**

Intravenous morphine titration (IVMT) is the French gold standard for opioid treatment in the emergency department (ED). Nebulized morphine titration (NMT) may represent an alternative without venous access, but it has not been adequately studied in adults. We test the hypothesis that NMT is at least as effective as IVMT to initially manage severe acute pain in the ED.

**Methods/design:**

We designed a multicenter (10 French EDs), single-blind, randomized and controlled trial. Adults between 18 and 75 years with visual analog scale (VAS) ≥ 70/100 or numeric rating scale (NRS) ≥ 7/10 will be enrolled. We will randomize 850 patients into two groups to compare two routes of MT as long as VAS >  30 or NRS >  3. In group A (425), patients will receive an initial NMT for 5–25 min associated with titration of an intravenously (IV) administered placebo of physiologic serum (PS). In group B (425), patients will receive IVMT plus nebulized PS placebo.

NMT is defined as a minimum of 1 and a maximum of 3 5-min nebulized boluses of 10 mg or 15 mg (weight ≥ 60 kg), at 10-min fixed intervals. IVMT is defined as a minimum of 1 and a maximum of 6 boluses of 2 mg or 3 mg (weight ≥ 60 kg), at 5-min fixed intervals. Nebulized placebo titration will be performed every 10 min. IV titration of PS will be performed every 5 min. In both groups, after 25 min, if VAS > 30/100 or NRS > 3/10, routine IVMT will be continued until pain relief.

Pain severity, vital signs, bronchospasm, and Ramsay score will be recorded every 5 min. The primary outcome is the rate of relief obtained 1 h from the start of drug administration. Complete pain relief in both groups will be compared with a non-inferiority design. Secondary outcomes are pain relief at 30 min (the end of NMT) and at 2 h and median pain relief. We will compare final doses, and study the feasibility and tolerance of NMT (protocol deviations, respiratory or hemodynamic depression, sedation, and minor vegetative side effects). Co-analgesia will be recorded. Discharge criteria from the ED and hospital are defined.

**Discussion:**

This trial is the first multicenter randomized and controlled NMT protocol for severe pain in the ED using the titration concept. We propose an original approach of combined titration with an endpoint at 1 h and a non-inferiority design.

**Trial registration:**

ClinicalTrials.gov, NCT03257319. Registered on 22 August 2017.

**Electronic supplementary material:**

The online version of this article (10.1186/s13063-019-3326-3) contains supplementary material, which is available to authorized users.

## Background

The treatment of severe acute pain in the emergency department (ED) has been a public health priority in France for several years [1–4]. Spontaneous pain is the primary reason for ED consultation, whatever the etiology [3]. Severe pain in the ED is defined by a visual analog scale (VAS) ≥ 60/100 or numeric rating scale (NRS) ≥ 6/10, and the French consensus conference on the subject [5–7] recommends emergency administration of intravenous morphine titration (IVMT) for pain relief. Morphine boluses of 2 or 3 mg can be administered every 5 min until relief is obtained (VAS ≤ 30 or NRS ≤ 3) [8–12]. Many studies have shown the safety and benefit of IVMT [13–16]; in particular, this approach has the advantage of being ubiquitous, while offering support for the concept of individualized dose-limiting side effects. The large intervariation and intravariation of the morphine molecule is thus taken into account, contrary to when dose-weight posology is administered. Side effects are known to be rare with IVMT: Respiratory depression associated with morphine is uncommon (or nil), and minor side effects do not exceed 10–15% [11, 12]. Other routes of morphine administration, titrated or not (oral, subcutaneous), do not allow such rapid relief, are not easy to handle safely, and are actually reserved for long-term treatment [5–9]. IVMT has become the standard morphine titration protocol in French EDs in cases of spontaneous severe acute pain [5–7].

Despite legal provisions and learned society recommendations, IVMT remains insufficiently applied or applied with delays [17]. The availability of morphine is good (low direct costs, low indirect costs of nursing administration and educational intervention), but the real requirements of opioids are still too limited, and oligoanalgesia persists in many countries [18–20]. Moreover, deviations from recommended protocols are frequent; i.e., additional doses are administered at abnormally long intervals between boluses and discontinued at an early stage [12]. Organizational causative factors are numerous, including the high workload especially in overcrowded EDs (significant flow of patients): there are restrictive legal conditions of deliverance as well as rigidity of application with the burden of implementation, and this method is particularly time-consuming for nurses. The need for rapid venous access is also negatively involved. It is difficult to dissociate blood sample tests for diagnosis purposes and venous therapeutic access for analgesia. To save time and to avoid a second puncture, nurses may wait for all prescriptions before setting up the venous access. Moreover, IV access is algogenic in itself, linked to added risk of infection and decreased mobility in the ED particularly in ambulatory patients. Nebulized opiates without venous access might represent another route of administration, providing management of opiates in cases of severe pain that could be practical for sites with limited means (prehospital as an inpatient) or sites that are maintained in substandard conditions.

However, data on the pain management of adults using a nebulized route in the ED are scarce. The majority of clinical studies involve patients in long-stay medical departments who have dyspnea and not pain (cancer or chronic respiratory insufficiency [21–26]). Some work has been carried out in pediatric EDs [27] or only in trauma patients [28–30]. Some protocols have tested inhaled morphine hydrochloride, and others fentanyl [27, 31]. Aerosol techniques vary from one study to another (intranasal administration, facial nebulization) as well as posology and pain intensity at inclusion (i.e., moderate and severe pain tested simultaneously). Criteria and time to pain assessment seem to be determined empirically (complete relief for patients, 50% reduction in pain intensity, etc.). Moreover, analysis of the literature shows that previous protocols involving nebulized morphine for pain relief in the ED aimed to evaluate a weight-associated “loading dose” rather than a morphine titration protocol. For example, a recent study tested an emergency dose of 0.2 mg/kg of morphine, unsuccessfully [28]. Although morphine is probably absorbed via the ear-nose-throat and bronchopulmonary systems, the variability of the pharmacodynamic effects of morphine for the same plasma concentration is unlikely to be modified [32–34] and remains a central parameter. Nebulized morphine titration (NMT) could be the alternative to IVMT (current reference treatment) with the same objectives, ensuring the most standardized and reproducible route of administration and the easiest method of controlling bioavailability and maintaining safety conditions in the ED. One study published in 2015 included 300 young trauma patients in the ED with the aim of testing two protocols of NMT, with two different bolus doses of morphine for repeated administration every 10 min [30]. In reality, the small number of boluses reported (1 is the median value for the highest dose versus 2 for the lowest dose) questions the morphine titration concept. In brief, the principle of morphine titration is most often forgotten or betrayed in the opioid nebulization protocols proposed in the literature, and comparative clinical trials with IVMT protocols are rare.

Pharmacokinetic data are also insufficient, even in healthy volunteers. Some data in healthy volunteers without any pain stimulus are available, but the results are not consistent and dyspnea seems to have been the priority area of research [35–40]. The nebulization durations are arbitrary, resulting in great variability in the kinetics of absorption of opiates tested and studies that are difficult to compare. The bioavailability measured varies from 8 to 59%, and the delay of the peak effect ranges from 0 to 10 min, sometimes comparable and sometimes preferable to oral administration. Absorption is sometimes described as an exponential model and sometimes as an inverse curve with immediate absorption between 50 and 75%. Some studies have attempted to specifically determine the links between the pharmacokinetics of opiates and the size of aerosolized particles [37, 38, 40–43] and/or the aerosol material, but the exact bioavailability of a morphine hydrochloride aerosol based on a nebulization system routinely used in a hospital wall socket remains poorly studied.

Considering the results reported in the literature and clinical practice, many questions remain before switching from IVMT to NMT. No simple conversion can be made between the 3-mg or 2-mg intravenous bolus, which is the reference for titration, and bolus doses to be nebulized. No preliminary study has determined which optimized and standardized dose of nebulized bolus could be repeated to achieve complete pain relief in patients and ensure safety and tolerance. The ideal times of the aerosol bolus and between each aerosol bolus have not been tested yet and cannot be deduced from what is proposed intravenously. Some clinical studies have tended to show relief, especially at the beginning of nebulization, in the first 5 min (when measured), and others have shown a greater rapidity of appearance of metabolites of morphine in the blood than in other routes of administration but without studying pain relief parallelism [27, 28, 35, 36]. Again, the data are insufficient and deserve to be completed.

In the absence of sufficiently robust data, further research on pain in healthy volunteers appears to be an essential prerequisite for a clinical trial on major emergencies. We performed AEROMORPH1 (EUDRACT 2013-001977-26, results in submission process) in order to determine, within a nebulized morphine protocol in healthy volunteers with pain (phase I trial), the pharmacokinetic characteristics and the feasibility of a single 5-min nebulized safety bolus of morphine which could be tested and repeated in the ED.

Given the AEROMORPH1 preliminary data and taking into account the poor results in the literature, we decided to propose the initiation of morphine titration by a new route of administration rather than exclusive NMT. We aim to test the hypothesis that a combined method of morphine titration, initiated by a maximum of 3 nebulized boluses, is at least as effective as IVMT performed exclusively according to the reference protocol for the treatment of patients with severe spontaneous pain (VAS between 70 and 100 mm or NRS ≥ 7/10) in the ED. We decided to perform a multicenter, randomized, placebo-controlled study, a methodological design associated with the highest level of evidence.

## Methods/design

### Study aims

The aim of our study is to evaluate two different methods of analgesia by morphine titration for the management of severe acute pain in the ED. First we will compare a combined morphine titration started by NMT and then IVMT, and second the reference route of administration of morphine titration, i.e., exclusive IVMT.

The primary outcome is to assess whether the two methods are as effective to provide pain relief at 1 h from the beginning of morphine titration.

Secondary outcomes are first, to compare for each titration method all the parameters of analgesic efficacy, second to assess the feasibility of this new method of combined morphine titration, and third to evaluate the tolerance of the nebulized route of morphine administration.

### Study design

#### Setting

**Fig. 1:**
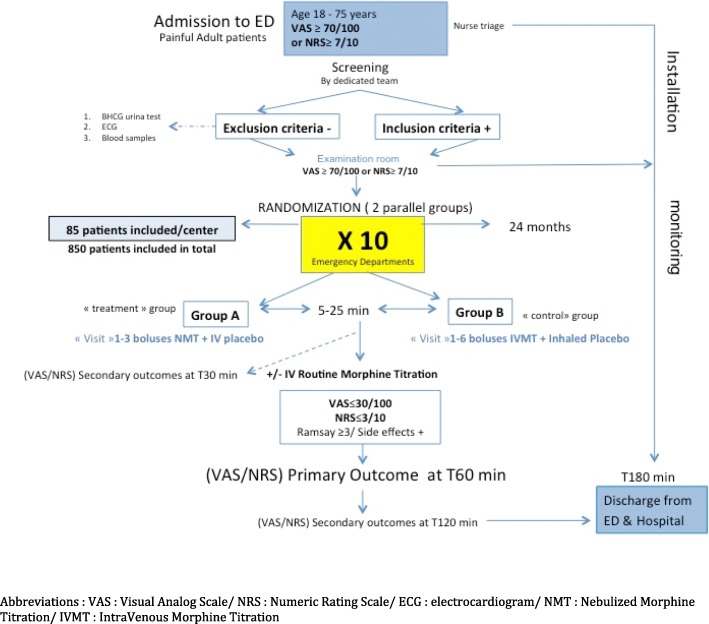
CLIN-AEROMORPH study flow chart

The CLIN-AEROMORPH trial is a multicenter, prospective, randomized, double-dummy, placebo-controlled trial conducted in 10 French adult EDs in tertiary care academic hospitals, with more than 30,000 patient visits per year. All these centers manage more than 2500 patients with severe pain per year routinely using IVMT according to French recommendations and have already participated in randomized controlled trials. Rouen University Hospital’s adult ED will coordinate the study and handle the data analysis. The coordinating investigator of this center and of the whole study has conducted large trials on IVMT before [12]. The trial will be conducted in the flow of routine patient care. A single-blinding design was the only possibility: Blinding may not be complete because of the aerosol preparation by nurses and physicians. The CLIN-AEROMORPH study design and flow chart are provided in Fig. 1.

#### Subject selection and inclusion criteria

Criteria for eligibility are adult patients, 18–75 years of age, presenting to the ED with a primary complaint of spontaneous severe acute pain who are being considered for admission to the ED observation unit whatever their pain etiology. An episode of severe pain is defined as the occurrence of pain that is rated 70/100 or more on the VAS (0–100), and when the VAS evaluation is impossible, pain that is rated 7/10 or more on the NRS (0–10). The VAS handheld slide rule will be shown and explained to the patients. When patients have difficulties in manipulating the VAS slide rule, nurses will be allowed to use the NRS (from 0 to 10), as these two scales are equivalent in the ED [5]. All nurses in the ED have been trained to assess pain using these unidimensional scales. A second VAS or NRS measurement will be taken in the examination room, to confirm the first measurement of severe acute pain recorded in the ED. If this second measurement confirms VAS ≥ 70/100 or NRS ≥ 7/10, the investigator will check the inclusion and exclusion criteria for enrollment.

Patients will be screened consecutively 5 days per week (Monday to Friday) during the day, when the organizational conditions for enrollment are met, until the sample size of 850 patients is reached. Patients cannot be consecutively enrolled in the study because high inflow or high workload in participating EDs is an obstacle, and is difficult to predict. Patients will be included when the study coordinator or a dedicated team member is available for enrollment and when a single examination room is available for treatment.

Inclusion and exclusion criteria are presented in Table 1.

**Table 1:** Inclusion and exclusion criteria

Inclusion criteria	
• Age ≥ 18 and < 75 years	
• VAS ≥ 70/100 or NRS ≥ 7/10	
• Patient affiliated with or beneficiary of a social security category	
• For women (childbearing age)	
○ Effective contraception (oral contraception, intrauterine device or use of condoms)	
○ Menopausal status (amenorrhea less than 12 months before the inclusion visit)	
○ Objectivized sterility (diagnosis or surgically)	
Exclusion criteria	
• Chronic pain (> 3 months)	
• Taking opioids more than 10 days (including opioids as tramadol and/or codeine)	
• Taking opioids in the emergency room within 4 h	
• Taking rifampicin	
• Impaired ability to discern, cognitive impairment	
• Morphine-related contraindications	
○ Chronic obstructive or restrictive respiratory failure known or suspected, compensated or not	
○ Hypersensitivity to the active substance or to any of the excipients	
○ Severe hepatocellular insufficiency (known or suspected)	
○ Chronic renal failure known or suspected	
○ Acute renal failure (creatinine clearance < 60 ml/min/1.73 m^2^ and/or increase of 25% from baseline)	
○ Uncontrolled epilepsy	
○ Cranial trauma (intracranial hypertension)	
○ Associations with buprenorphine, nalbuphine, pentazocine, or naltrexone	
• Active drug history or practice(s)	
• Obvious need to reduce fracture or dislocation in the emergency room	
• Suspected occlusive syndrome	
• SpO2 < 95%	
• Respiratory rate (RR) < 12 breaths/min	
• Glasgow Coma Scale (GCS) < 15 or other alertness disorders	
• Heart rate (HR) < 50 bpm and/or auriculo-ventricular block (PR interval > 200 ms)	
• Arterial hypotension with systolic blood pressure TA syst < 100 mmHg	
• Pregnancy or lactation	
• Persons deprived of their liberty by an administrative or judicial decision, persons placed under the safeguard of justice, guardianship	
• Patients with poor comprehension of spoken or written French	
• Patients participating in another interventional clinical study	
• Contraindication related to the use of saline solution	
• Contraindications related to the use of aerosol:	
○ Necessity to access the face	
○ Allergy known to plastic	
○ Claustrophobia	

#### Randomization, allocation concealment

Informed consent may be waived at randomization, because patients may need urgent pain management and because acute pain impairs the ability to provide informed consent. Whenever a patient is included without written informed consent, such consent will be promptly sought from the patient when the pain has decreased, according to the French Law of Ethics. However, each patient will be introduced to the trial by a member of the research group and will receive an explanation of the study protocol (not including random assignment of the morphine treatment) before the senior ED physician in charge of the patient can obtain appropriate written informed consent.

The randomization list will be generated before commencement of the study. We will use computer generated random numbers to generate the allocation sequence, without blocking. Patients will be randomized into one of two parallel groups stratified by sex and center using centralized software (Clinsight®, Ennov Group, Paris, France). In arm A (*n* = 425), patients will receive first, a combined morphine titration started by NMT plus IV placebo of physiologic serum (PS) for the first 25 min and then second, routine IVMT in case of persistent pain after 25 min of treatment as standard of care in the ED. Patients in arm B (*n* = 425) will receive IVMT and a nebulized placebo of PS during the same time (25 min). If the VAS score is > 30 or the NRS score is > 3 after the maximum number of IVMT boluses, routine IVMT without nebulized placebo will be performed until pain relief as standard of care in the ED.

### Study procedures and intervention

#### Treatments

The aerosol material (aerosol mask, plastic tubing, and PVC transparent tank) used will be comparable to the material routinely used in ED for other drug administrations like terbutaline in asthma (CE0120 marking - Class IIa). It allows the nebulization of drugs with an average mass diameter of 3.6 μm, at a rate of 0.25–0.3 ml/min. The nebulization technique proposed was tested in our previous study on healthy volunteers, AEROMORPH1. In arm A, morphine titration will be done by repeated nebulized bolus administration of morphine hydrochloride, using 10 mg (patient’s weight < 60 kg) and 15 mg (patient’s weight ≥ 60 kg). A morphine solution will be reconstituted by dilution with PS so that the final volume will be reduced to 3 ml in the aerosol mask tank. The morphine will be nebulized for 5 min at a constant airflow of 10 L/min, and 3 ml of PS placebo will be administered by intravenous push at the beginning and at the end of each aerosol. In arm B, IV morphine will be administered by intravenous push, using 2 mg (patient’s weight < 60 kg) or 3 mg (patient’s weight ≥ 60 kg) as recommended by French guidelines on acute pain management [5–7]. At the same time, 3 ml of PS placebo will be nebulized for 5 min at a constant airflow of 10 L/min.

Specific treatments required in case of adverse events (AEs) are described in the protocol. In case of severe ventilatory depression (respiratory rate (RR) < 10 breaths per minute), naloxone titration (intravenous bolus of 0.04 mg) will be administered until RR is more than 12 breaths per minute see see (Additional file 1). In case of vomiting, IV ondansetron will be started at a dose of 4 mg; ondansetron is a central action molecule that has shown efficacy in morphine-dependent side effects. IV metoclopramide will only be used in case of failure, at a dose of 0.5 mg/kg (100 ml bag infusion over 15 min). In case of failure of the two previous treatment lines, dexamethasone will be injected at a dose of 4 mg.

#### Study protocol

**Fig. 2:**
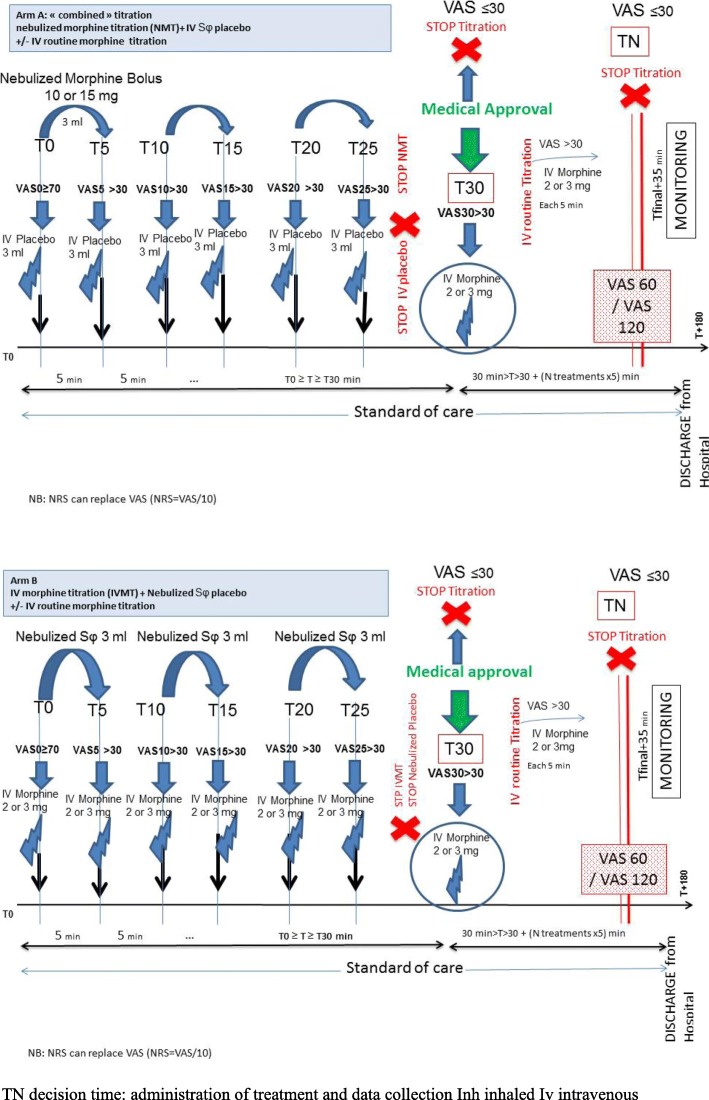
Study design

This protocol has defined the dose of IV or nebulized boluses of morphine, the interval between boluses, the absence of limitation of the total dose, the VAS threshold required to administer morphine, and the criteria to stop titration. All nurses in the ED have been trained to perform morphine titration. A specific form is used for data collection see (Additional file 2). Drug administration will begin at time 0 and finish at T25. The schedule of the study is described in Fig. 2. Morphine titration will be administered until a VAS score of ≤ 30 (NRS ≤ 3) is reached, or until the onset of a serious AE. When the patient is asleep, no attempt will be made at arousal. In this situation the patient will be considered as having adequate pain relief.

**Fig. 3:**
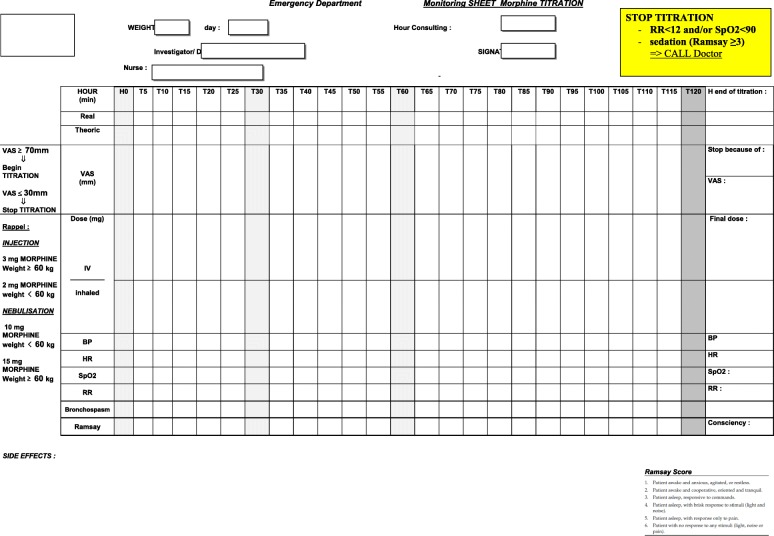
Monitoring sheet

The patient’s pain score will be recorded in the patient’s case report using a Monitoring Sheet (Fig. 3) at time points 0, 5, 10, 15, 20, 25 30, 60, and 120 min by a single investigator. After the end of 180 min of monitoring, the study will be terminated. In arm A, nebulized morphine will be administered with a minimum of 1 and a maximum of 3 5-min boluses of 10 mg (weight < 60 kg) or 15 mg (weight ≥ 60 kg), at 10-min intervals, for 5–25 min. During this period, 1 to 6 IV placebo boluses will be required every 5 min. In arm B, IV morphine will be administered with a minimum of 1 and a maximum of 6 boluses at 5-min intervals (as recommended by the French guidelines on acute pain management [5–7]) for 5–25 min. During this period, 1 to 3 nebulized placebo boluses will be required at 10-min intervals, for 5 min. In both arms, if pain relief is not reached at 30 min (T25 + 5 min), routine IVMT will be performed after validation by the ED physician. No maximum dose will be imposed. The final stopping criteria are the same as previously described.

**Fig. 4:**
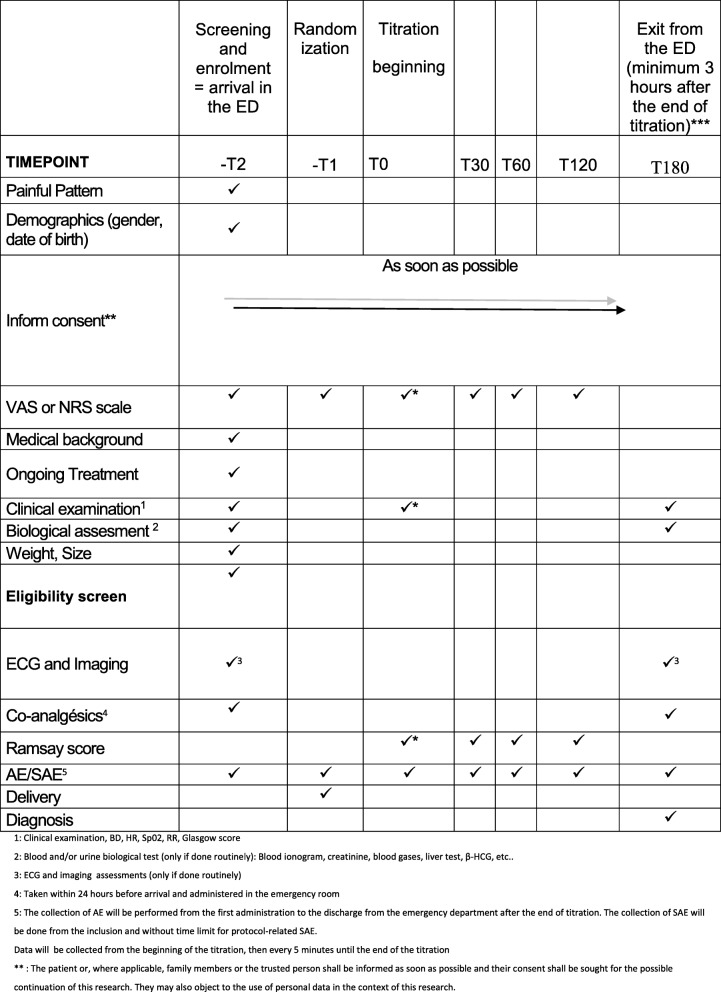
Schedule of enrollment, interventions, and assessments

Discharge from the ED will be allowed 1 h after the end of titration, except for radiological examination or transfer inside hospital, and discharge from the hospital will be organized 3 h after the end of titration (6 h in case of unexpected renal failure). An information sheet will be delivered on the risks of morphine titration (urinary retention, constipation) after the visit. Clinical monitoring will include RR measurements, pulse oximetry (SpO2), sedation according to Ramsay score [12], arterial blood pressure, and heart rate. Morphine titration will be stopped if the patient has an RR less than 12 breaths per minute and/or SpO2 less than 90%, Ramsay score ≥ 3, and/or a serious AE related to morphine administration as previously described. The schedule of enrollment, interventions, and assessments is presented in Fig. 4. Safety procedures are provided in case of respiratory depression see (Additional file 1).

### Outcome parameters

The primary outcome of this study is complete pain relief, related to the final VAS (0–100 mm) or NRS (0–10). The primary endpoint is the relief rate defined as VAS ≤ 30/100 or NRS ≤ 3/10 at 1 h from the beginning of titration.

Secondary outcomes are to compare all the parameters of analgesic efficacy for each method of titration and to evaluate the feasibility and tolerance of the nebulized route of morphine administration.

Analgesic efficacy will be measured by assessing the total dose of morphine administered (based on weight and body mass index (BMI)), the median delay of relief, the proportion of patients relieved according to the duration of titration (pain relief at 30 min and 120 min from the start of titration), and the proportion of patients totally relieved by exclusive NMT. Feasibility will be evaluated by measuring the rate of deviation from the protocol (major and minor; see Table 2). For side effects, we will record the incidence of respiratory depression, the incidence of sedation by the Ramsay score [44, 45], and the incidence of vegetative minor side effects associated with opiates (nausea, vomiting, urinary retention).

**Table 2:** Morphine titration protocol deviations

Error	Effect	Deviation
RR not taken 1 time	Underestimated	Minor
RR not taken at least 2 times	Underestimated	Major
RR < 12 and bolus done	Underestimated	Major
RR > 12 and bolus stopped	Overestimated	Major
Uncoded SpO2	Underestimated	Minor
Uncoded Ramsay	Underestimated	Major
Continued at Ramsay > 2	Underestimated	Major
Uncoded side effects	Underestimated	Minor
Side effects and bolus pursuit	Underestimated	Major
Too high dose, weight < 55 kg	Overestimated	Major
Too high dose, weight 55–60 kg	Overestimated	Minor
Too low dose, weight 60–65 kg	Underestimated	Minor
Too low dose, weight > 65 kg	Underestimated	Major
Stopped at 35 ≤ VAS < 40 not related to side effects	Underestimated	Minor
Continued at VAS ≤ 25	Overestimated	Minor
Started at 65 ≤ VAS ≤ 70	Overestimated	Minor
Stopped at VAS ≥40 not related to side effects	Underestimated	Major
Continued at VAS ≤ 20	Overestimated	Major
Started at VAS ≤ 60	Overestimated	Major
Time between bolus 5 < *T* ≤ 7 min	Underestimated	Minor
Time between bolus 3 ≤ *T* ≤ 5 min	Overestimated	Minor
Time between bolus > 7 min	Underestimated	Major
Time between bolus < 3 min	Overestimated	Major
Nebulized bolus dose # 10 or 15 mg	Exclusion	Major
IV bolus dose # 2 or 3 mg	Exclusion	Major
No times recorded	Exclusion	Major
Refusal of the patient not respected	Exclusion	Major

Abbreviations: *min* minutes, *mg* milligrams, *RR* respiratory rate, *#* different, *VAS* visual analog scale

In addition, a description of co-analgesia will be provided: nature, dose, time to start. Co-analgesics are analgesics administered up to 24 h before the emergency room consultation, concomitant analgesics, and analgesics administered after morphine titration and up to 2 h after its completion.

### Usual care

For patients randomized in both arms, the attending physician will perform the usual clinical examination, and therapeutic treatments will be administered. The choice of drugs and dose will be left to the discretion of the ED physician, as previously reported. Additional analgesia can be administered before, during, or after morphine titration for multimodal pain relief.

### Data collection

Clinical-biological data will be entered in the secure online database of Rouen University Hospital, using Clinsight® software (Ennov Group). The data will be hosted on a secure server located in the Information System Department of CHU-Hôpitaux de Rouen (Rouen University Hospital). Backup copies of the contents of this server are made daily. The data will be entered by the investigator or the clinical study technician of each center, who will have personal and secured accounts (username and password personalized to 6 characters minimum) to access the database of the study. The person carrying out the data processing (biostatistician) will have access to the data once the base has been frozen on a secure server accessible only to the data manager and to the biostatistician of the study.

### Statistical methods

The purpose of this study is to establish the non-inferiority of nebulized morphine titration as compared to IV morphine for relief of severe pain events in adults in the ED. The study is a repeated measures study, measuring pain scores before, during, and after treatment. The desired primary clinical outcome is that nebulized morphine titration is at least as effective in relieving pain as IV morphine 1 h after administration of medication.

In keeping with the non-inferiority nature of the test, a one-sided test at the nominal 0.05 significance level will be performed based on test statistic *z*. This analysis will be complemented by an adjusted analysis based on the linear model in order to adjust treatment comparisons for center and potential factors associated with complete pain relief. In order to obtain 80% power to reject non-inferiority for a 7% margin, and provided there is no difference between true proportions of complete pain relief (i.e., πiv = πae), the required sample size is 404 patients per arm, i.e., 808 patients overall (using software nQuery Advisor, version 6). Owing to the per protocol nature of the main analysis, and in order to account for the possibility of failure to completely or properly administer morphine (i.e., according to planned schedule), the target sample size is 850 patients overall in order to obtain 808 patients in the main per protocol analysis.

Regarding secondary outcome criteria, comparative analyses between the two treatment arms will be performed in an intention-to-treat analysis. Comparisons will be based on Student’s *t* test for quantitative variables, Pearson’s chi-square test for categorical variables, or the logrank test for time-to-event variables.

We will provide a descriptive account of the two treatment groups at baseline in terms of demographics, recruiting center, and baseline values of all study outcomes. All patients and carers will be analyzed according to the group to which they were randomized. No interim analysis is planned.

### Bias and confounding variables

In terms of selection bias, we consider that this study targets a patient population to whom this research ultimately will be clinically applicable and valuable. The trial will be conducted in the flow of routine patient care. This approach will enable us to test morphine titrations in the real-world setting, giving high external validity to the results. Every effort will be made to ensure that recruitment of participants occurs 5 days per week, as patients will be recruited by the ED physician treating the patient, but inclusion will be done by another physician. Co-analgesia will be more evaluable due to this procedure. We anticipate that the randomized, controlled design of this study will minimize the effect of confounding variables on our analysis. The bias linked to the single-blind design is obvious, but the feasibility of this study at the bedside justifies this methodological choice. The bias linked to missing data is reduced because there is a dedicated research team in charge of the study in most of the EDs.

### Safety reporting

All AEs that occur during the study period observed by one of the clinical staff, or reported by the patient or parent/guardian spontaneously or in response to a direct question, will be noted on appropriate forms, i.e., for AEs, serious adverse events (SAEs), or suspected unexpected serious adverse (drug) reactions (SUSARs). AEs will be classified on the form in terms of their severity, association with the study drug, expectedness, and seriousness. They will be recorded on an AE log. AEs will be reported to the sponsor as soon as possible and on a yearly basis as part of an annual safety report and at the end of the trial. The sponsor (CHU-Hôpitaux de Rouen/Rouen University Hospital, Department of Research and Innovation) has the responsibility to ensure all relevant and available information is forwarded to the competent authority (Irish Medicines Board) and the appropriate health ethics committee. It is important to note that a nurse and a physician will be immediately available (in less than 1 min) to manage any possible side effects during the study time.

An independent monitoring committee (ICS) of three experts has been constituted to analyze and classify the critical events and their possible link with opiates, especially death, respiratory depression, and uncontrollable vomiting that do not respond to treatment plans. A committee of three independent external expert investigators will be established. This committee will monitor the study and may be requested at any time by the sponsor to issue a written report on accountability in any SAE processing opinions or data appearing in the study that are likely to change the risk/benefit ratio of the research. The committee will meet at least once per year and whenever necessary upon request of the sponsor. Written reports of meetings of the expert committee will be forwarded to the sponsor and investigators. The annual safety reports will be given to each member.

## Discussion

This protocol will investigate an alternative route of administration of morphine to reduce the time delay to relief, without losing the benefits of titration. Previously, we had to manage with the poor results reported in the literature. This lack of available data led us to propose first a study protocol based on two different bolus doses of morphine, 10 mg or 15 mg, linked to body weight. According to a recent study [30], the safety of a nebulized bolus of up to 20 mg now seems established, which allowed us to compare these lower doses.

Regarding the primary endpoint of our study, we will measure the main outcome at 1 h for both pharmacokinetic and ethical reasons. First, this outcome is the same as that of our large study on IVMT in the ED [12] with the objective of complete relief, related to VAS ≤ 30/100 or NRS ≤ 3/10. This previous study established that when IVMT is performed in the ED for spontaneous severe acute pain, the median number of boluses expected is 3 (95% confidence interval (CI), 3–4), but with a range between 1 bolus and more than 10 boluses [12]. Moreover, our study protocol is original given the “combined” design of arm A, which means that IVMT is not a rescue procedure but a real part of the protocol, before this main outcome endpoint at 1 h (IVMT begins at 30 min). The objective is to replace only the first 5 IV boluses of morphine, not to replace or shorten the entire IVMT. Evaluating criteria during the titration process should be avoided. Our primary outcome at 1 h is a consequence of these pharmacokinetic considerations. Secondly, this evaluation at 1 h seems to be ethically acceptable in case of failure.

Incidentally, our study protocol shows many differences with the Grissa et al. study [30], such as the shorter duration of the aerosol application, a time lapse without treatment (5 min), and in the control group, a standard of care that conforms more to the French learned society recommendations with IVMT doses adjusted to weight ≥ 60 kg. Finally, the Grissa et al. study aimed for a 50% reduction in pain intensity, while we preferred to target total pain relief, but with combined titration. We chose an endpoint with the same delay, but we chose to perform a non-inferiority trial to study the complete relief of severe acute pain using this new combined NMT + IVMT method of titration.

### Trial status

Patient enrollment started on 19 September 2017 after Ethics Committee approval. The end of enrollment is scheduled for 19 September 2019.

## Additional files

Additional file 1: Naloxone protocol. (DOC 25 kb)

Additional file 2: Standard Protocol Items: Recommendations for Interventional Trials (SPIRIT) 2013 checklist. (DOC 132 kb)

## Abbreviations

AE: Adverse event
BMI: Body mass index
ECG: Electrocardiogram
ED: Emergency department
GCS: Glasgow Coma Scale
HR: Heart rate
IMC: Independent monitoring committee
IV: Intravenous
IVMT: Intravenous morphine titration
MT: Morphine titration
NMT: Nebulized morphine titration
NRS: Numeric rating scale
PS: Physiologic serum
RR: Respiratory rate
SAE: Serious adverse event
SOP: Standard operating procedure
SUSAR: Suspected unexpected serious adverse (drug) reaction
VAS: Visual analog scale
