# Supplementary material for: Nebulized versus intravenous morphine titration for the initial treatment of severe acute pain in the emergency department: study protocol for a multicenter, prospective randomized and controlled trial, CLIN-AEROMORPH
Source: Trials. 2019 Apr 11;20:209. doi: 10.1186/s13063-019-3326-3 (PMC6458825; doi:10.1186/s13063-019-3326-3)
Supplement: Supplementary file 1 — Naloxone protocol. (DOC 25 kb) [file 13063_2019_3326_MOESM1_ESM.doc]

Additional file 1

**Protocol Naloxone [12]**• Naloxone bolus 0.4 mg / 1 ml
• Indications: if RR ≤ 9, despite stopping administration of morphine
• Methods of administration: Dilute 1 bolus in 10 cc water PPI-Inject ml / ml IVD every 2 min until a RR> 12/mn
• Onset: 2 min: reassessment of the RR
• Duration of action: 20-30 min
• Monitoring for 4 to 6 hours.
If mechanical ventilation is required despite this protocol, ICU treatment will be provided.

Abbreviations: RR Respiratory Rate/ min minutes/ ICU Intensive Care Unit
